# Neuroendocrine patterns underlying seasonal song and year-round territoriality in male black redstarts

**DOI:** 10.1186/s12983-021-00389-x

**Published:** 2021-02-24

**Authors:** Camila P. Villavicencio, Harriet Windley, Pietro B. D’Amelio, Manfred Gahr, Wolfgang Goymann, René Quispe

**Affiliations:** 1 Department of Behavioural Neurobiology, Max Planck Institute for Ornithology, Eberhard-Gwinner-Str. 6a, D-82319 Seewiesen, Germany; 2 Department of Ecological Science, Institute of Ecology and Biodiversity, Faculty of Science, University of Chile, Las Palmeras 3425, Ñuñoa, Santiago, Chile; 3 FitzPatrick Institute of African Ornithology, University of Cape Town, Rondebosch, 7701 South Africa; 4 Department of Marine Biology, Faculty of Ocean Sciences, Universidad Catolica del Norte (UCN), Larrondo 1281, Coquimbo, Chile

**Keywords:** Sexual hormones, *Phoenicurus ochruros*, Brain receptors, Hypothalamus, Song control system, Aggressive behavior, Seasonality, Individual variation, mRNA expression, In situ hybridization

## Abstract

**Background:**

The connection between testosterone and territoriality in free-living songbirds has been well studied in a reproductive context, but less so outside the breeding season. To assess the effects of seasonal androgenic action on territorial behavior, we analyzed vocal and non-vocal territorial behavior in response to simulated territorial intrusions (STIs) during three life-cycle stages in free-living male black redstarts: breeding, molt and nonbreeding. Concurrently, we measured changes in circulating testosterone levels, as well as the mRNA expression of androgen and estrogen receptors and aromatase in the preoptic, hypothalamic and song control brain areas that are associated with social and vocal behaviors.

**Results:**

Territorial behavior and estrogen receptor expression in hypothalamic areas did not differ between stages. But plasma testosterone was higher during breeding than during the other stages, similar to androgen receptor and aromatase expression in the preoptic area. The expression of androgen receptors in the song control nucleus HVC was lower during molt when birds do not sing or sing rarely, but similar between the breeding and the nonbreeding stage. Nevertheless, some song spectral features and the song repertoire differed between breeding and nonbreeding. Territorial behavior and song rate correlated with the expression of steroid receptors in hypothalamic areas, and in the song control nucleus lMAN.

**Conclusions:**

Our results demonstrate seasonal modulation of song, circulating testosterone levels, and brain sensitivity to androgens, but a year-round persistency of territorial behavior and estrogen receptor expression in all life-cycle stages. This suggests that seasonal variations in circulating testosterone concentrations and brain sensitivity to androgens is widely uncoupled from territorial behavior and song activity but might still affect song pattern. Our study contributes to the understanding of the complex comparative neuroendocrinology of song birds in the wild.

**Supplementary Information:**

The online version contains supplementary material available at 10.1186/s12983-021-00389-x.

## Background

In many temperate species, male birds sing and establish a territory at the onset of the breeding season when circulating levels of testosterone are high (e.g. [1, 2]). Thereby, testosterone can influence several reproductive traits, including vocal and non-vocal territorial behavior [3–5]. Androgenic control of vocal and non-vocal territoriality is more evident in species that are territorial and sing only during the breeding season. However, there is growing evidence that circulating testosterone can be decoupled from territorial and song behavior [6–8]. In some species, plasma testosterone may facilitate vocal and non-vocal territorial behavior during breeding, but not outside the breeding season [9–12], especially in species that sing year-round or during extended periods [7, 13]. Further, in year-round territorial birds, territorial behavior can be independent from circulating testosterone even during breeding [14–17]. Therefore, the relationship between circulating testosterone and territorial behaviors can vary among species with different life histories, and within species among different life-cycle stages.

The neuroendocrine control of territoriality and song in songbirds has been associated with discrete brain areas that express androgen and/or estrogen receptors [18–20]. This implies that not only changes in the circulating levels of testosterone or estradiol, but also differences in the brain’s sensitivity to androgens and estrogens can affect reproductive behaviors [7, 21]. Oscine songbirds possess androgen-sensitive brain regions that are part of a network of forebrain nuclei involved in the learning and production of song [18, 22–26]. In addition, seasonal changes in aggressive and territorial behavior may depend on the conversion of testosterone into estrogens by aromatase (estrogen synthase) in brain regions that include hypothalamic areas, such as the preoptic area and the posterior hypothalamus [21, 27–29]. The preoptic area has also been related to the motivation to sing and thus may influence the song rate of songbirds [30, 31]. Therefore, studying seasonal changes of gonadal hormone receptors and aromatase activity in regulatory brain areas can provide important insights about the seasonal control of reproductive behaviors, including song and territoriality [14, 18, 23, 24, 32–34]. Variation of territorial behavior in birds rarely correlates with levels of circulating testosterone, possibly indicating an individual threshold function for the activation of such behavior (e.g. [35, 36]). The cellular and molecular properties of target brain areas may be key to understanding such variation in hormone action [37, 38]. Although seasonal modulation of circulating testosterone levels has been reported for many temperate and some tropical bird species (e.g. [1]), only few studies have evaluated the seasonality of hormone sensitivity of brain control areas in free-living songbirds [20, 34, 39]. Even fewer studies have investigated the seasonal variation of neural hormone sensitivity in combination with circulating hormone levels and related this variation to the expression of socio-sexual behaviors during different life-cycle stages [40].

We studied a central European migratory population of black redstarts (*Phoenicurus ochruros*), which winters in the Mediterranean and arrives at its’ breeding grounds further north in March. Upon arrival males establish territories for breeding [41]. Black redstarts continue to defend these territories after breeding from September until November when they start migrating to their wintering grounds. In late summer (August), when they molt, males generally do not sing [42], but it has been unclear if they still defend their territories during molt. Territoriality of black redstarts consists of an array of non-vocal behaviors including spatial behaviors (e.g. latency to approach an intruder, time spent close to the intruder, closest approach), threat displays (e.g. head nodding), and direct aggression (e.g. attack), together with vocal behaviors (song). All of these behaviors may be regulated by different neuroendocrine pathways [43]. Previous evidence suggests that there is no association between circulating levels of testosterone and non-vocal aggressive behaviors [8, 43, 44]. However, some spectral features’ of song seem to depend on circulating testosterone during breeding [45].

To better understand testosterone-mediated effects on territoriality across life-cycle stages, we analyzed non-vocal and vocal territorial behaviors of free-living male black redstarts in response to simulated territorial intrusions during three life-cycle stages: breeding, molt and nonbreeding (autumn territoriality). Concurrently, we measured the expression of androgen receptors (AR), estrogen receptors α (ER α) and aromatase (ARO) in specific brain areas in concert with circulating testosterone concentrations of males. In black redstarts, AR, ER α, and ARO expression has been reported in the preoptic area (POA), the posterior hypothalamic area (H), and two nuclei of the song control system, the HVC (proper name) and the lateral magnocellular nucleus of the anterior nidopallium (lMAN) during breeding and autumn [14]. However, the seasonal differences in receptor expression in these brain areas, including during the molting stage, remained to be explored. Hypothalamic preoptic regions are relevant for the control of sexually-motivated behavior [46] including the motivation to sing [30], and posterior hypothalamic areas associated with the expression of territorial and aggressive behaviors [28]. The HVC is involved in vocal motor behavior [4, 47], and it may influence various parameters of song patterns, including the control of spectro-temporal features (reviewed by [43]). lMAN activity is involved in song variability [48], which can be influenced by social context [49], and seasonal song [50].

We first asked if the behavioral and neuroendocrine traits vary among life-cycle stages. Because the HVC and the song control system regulate songs’ spectro-temporal features, we analyzed – for the first time in this species – the song repertoire. Second, because we measured behavioral data, plasma hormone levels and brain receptors in the same individual, we asked whether behavioral traits change as a function of circulating levels of hormones, and/or the neural sensitivity to these hormones. We predicted that if hormones regulate territorial behavior, changes in testosterone and/or androgen brain sensitivity will mirror the behavioral traits on a seasonal basis.

## Results

### Territorial behaviors

**Fig. 1:**
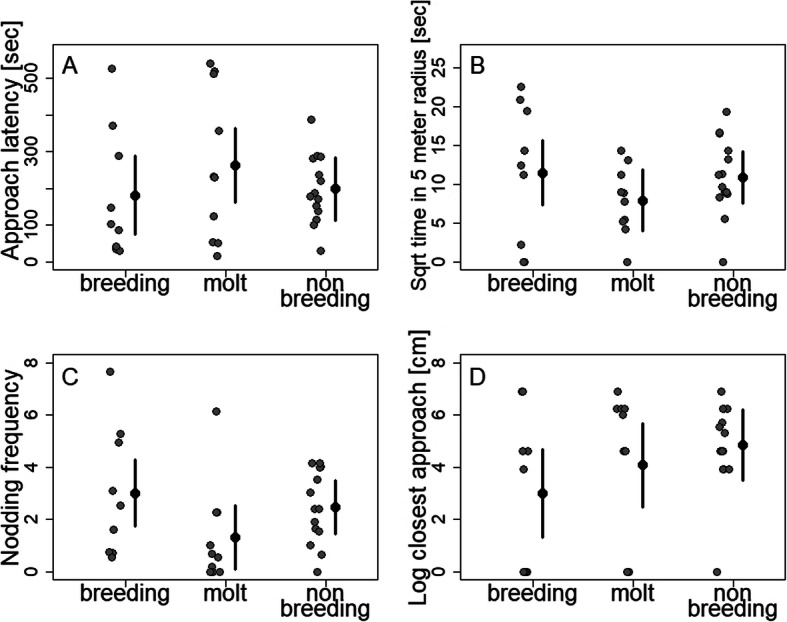
Male behaviors measured in response to a simulated territorial intrusion (STI) across seasons. Mean estimates ± 95% CI are shown together with individual data points. The latency of males to approach to the decoy (**a**), the time they spent in a five-meter radius around the decoy (**b**), the head nodding frequency (**c**), and the closest approach to the decoy (**d**) did not differ between breeding (*N* = 9), molt (*N* = 10) and nonbreeding (*N* = 14)

Non-vocal territorial behaviors measured during the STIs were similar between seasons (Fig. 1): Approach latency (F _(2, 30)_ = 0.75, *p* = 0.48, *R*^2^ = − 0.01), time spent in a five meter radius around the decoy (F _(2, 30)_ = 0.99, *p* = 0.38, *R*^2^ = − 0.0004), nodding frequency (F _(2, 30)_ = 2.06, *p* = 0.15, *R*^2^ = 0.06), and closest approach (F _(2, 30)_ = 0.45; *p* = 0.64, *R*^2^ = − 0.04) did not differ between seasons. However, males attacked the decoy only during breeding (3 out of 8 males) and not during the other seasons. Males that did not approach the decoy sang at a higher rate than males that approached the decoy (F _(1, 10)_ = 9.216, *p* = 0.013, *R*^2^ = 0.4).

### Song behavior

**Fig. 2:**
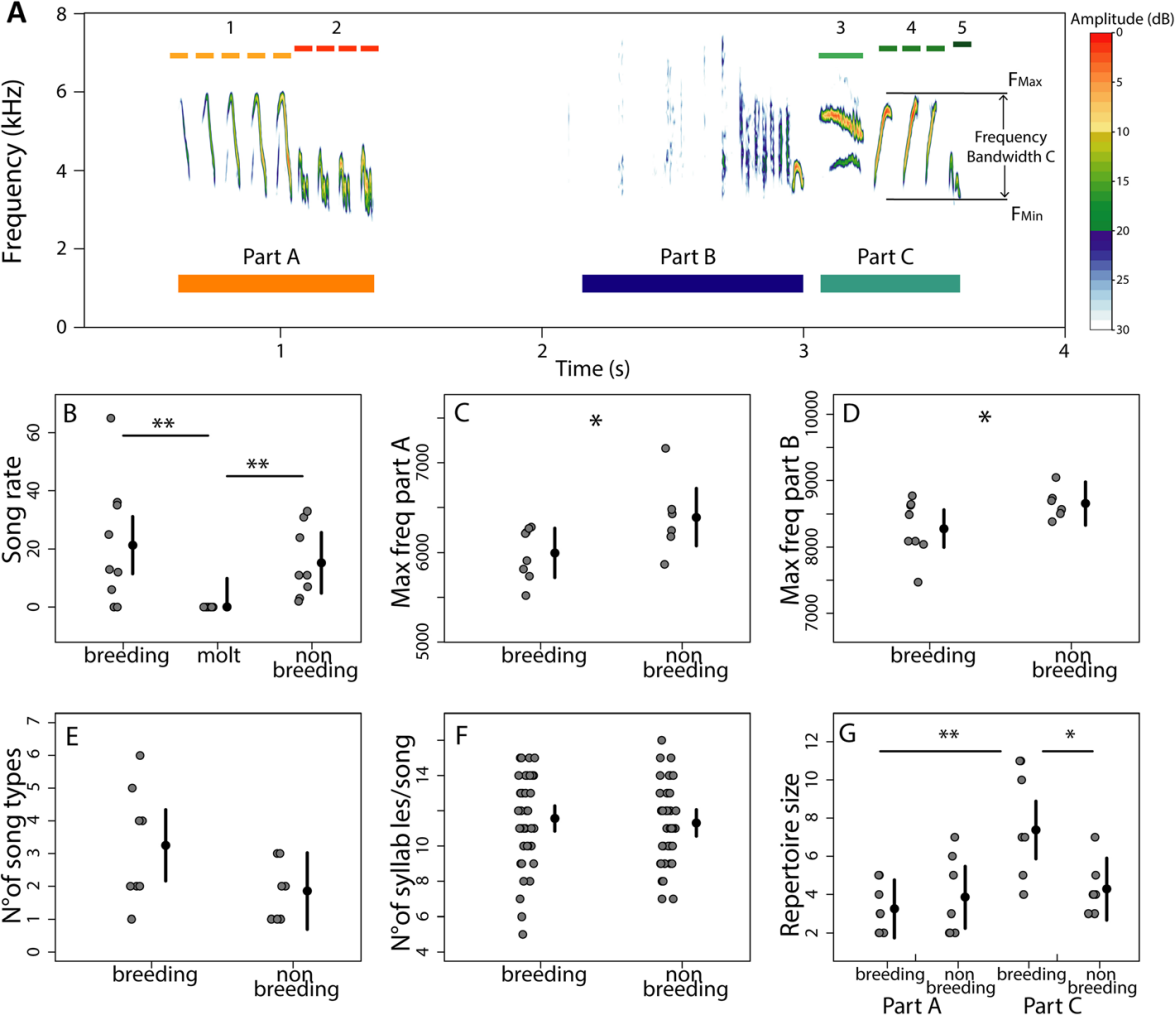
Male black redstarts’ song spectrogram and analysis. A whole strophe (**a**) of a male black redstart song (Spectrogram: R package “seewave” [51], sample rate 22, 050 Hz, FFT = 512 points, Hanning-Window, overlap: 70%), illustrating the structural components of the song: Part A, Part B and Part C. The syllable type (1-2-3-4-5) and the syllable division are shown in the upper part, for example, syllable type 1 has 5 elements. Part B is a single atonal element. Spectrogram also shows the pause between Part A and Part B and how acoustic frequencies were defined. The mean estimates ± 95% CI and data points of song rate (**b**), song features (**c**, **d**) and song repertoire (**e**, **f**, **g**) analyses across seasons are shown in the lower panels. Song rate 10-min after the STI (**b**) did not differ between breeding (*N* = 9) and nonbreeding (*N* = 8; *p* = 0.5), and both were significantly higher compared to molt (*N* = 8; *p* = 0.01, *p* = 0.008). The maximum frequencies of part A (**c**) and part B (**d**) were lower during breeding (*N* = 8) compared to nonbreeding (*N* = 6; *p* = 0.04, *p* = 0.05). The maximum frequency of part C (not shown) had a similar tendency but without significant difference. The number of song types (**e**) and the number of syllables per song (**f**) did not significantly differ between stages. The repertoire size (**g**) of part C was larger during breeding (*N* = 8) compared to nonbreeding (*N* = 7; *p* = 0.047) and larger than the repertoire size of part A during breeding (*p* = 0.01). Individual data are shown for all the panels. For the max. frequency of part A and B the individual mean of 20 strophes is shown. Asterisks indicate significance (* = *p* < 0.05, ** = *p* < 0.01)

The song rate of the 10-min period after the STI differed between seasons (F_2,24_= 5.3, *p* = 0.01, *R*^2^ = 0.25; Fig. 2b), being lower for molt compared to breeding (*p* = 0.01, Cohen’s d =1.3) and nonbreeding (*p* = 0.008, Cohen’s d = 1.6) and without significant difference between breeding and nonbreeding (*p* = 0.5, Cohen’s d =0.3). During the molting period only one male sang in response to the STI. Since we could not catch this male to verify the molting stage (1 out of 10), we did not include the song of this male in the song analyses. Therefore, all song comparisons were done between breeding and nonbreeding birds.

In the analysis of spectral features, we found that the maximum frequencies of part A and part B were lower during breeding than nonbreeding (Part A: χ^2^ =4.24, *p* = 0.039; part B χ^2^ =3.77, *p* = 0.05, Fig. 2c, d), and the maximum frequencies of part C, although showing a similar tendency, did not differ significantly between seasons (χ^2^ =1.7, *p* = 0.2). The minimum frequency of part C tended to be lower during breeding than nonbreeding (χ^2^ =3.4, *p* = 0.06). The pause between part A and part B and the duration of the song were shorter during breeding compared to nonbreeding (pause: χ^2^ =3.9, *p* = 0.048, song duration: χ^2^ =3.8, *p* = 0.049). All other parameters measured (maximum frequency of part C, minimum frequencies of part A and B, total frequency bandwidth, duration of part A, B and C, and overall frequency bandwidth of each entire strophe) did not differ between seasons (all p’s > 0.12).

In the song repertoire analysis, the number of song types and the repertoire size did not differ between stages (song type: F_1,13_ = 3.57, *p* = 0.08, *R*^2^ = 0.2; repertoire size: F_1,13_ = 2.65 *p* = 0.1, *R*^2^ = 0.1), although there was a tendency for both features to be higher during breeding (Fig. 2e). The number of syllables per song did not differ between breeding and nonbreeding (F_1,38_ = 0.23, *p* = 0.6, *R*^2^ = − 0.01, Fig. 2f). When analyzing the repertoire size of part A and part C separately (see Fig. 2a), the repertoire size in part A did not differ between breeding and nonbreeding, but in part C birds sang more syllable types during breeding than nonbreeding (*p* = 0.047). In addition, during nonbreeding the repertoire size was similar between part A and part C, but during breeding males sang more syllable types in part C compared to part A (*p* = 0.009, Fig. 2g). In summary, we did not find striking differences between breeding and nonbreeding songs, but there were subtle differences in the spectral features and the song structure (Fig. 2).

### Testis size and testosterone concentration

**Fig. 3:**
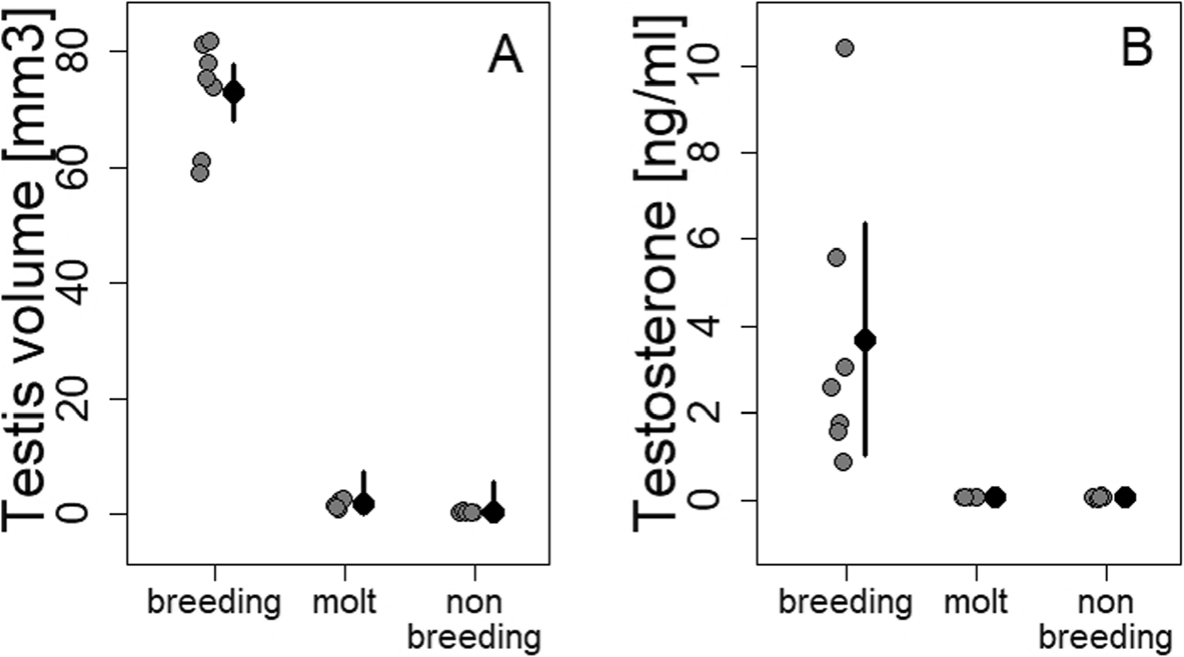
Mean estimates ± 95% CI and individual data points of testis volume and testosterone concentrations across seasons. Testes (**a**) were larger during breeding (*N* = 7) compared to molt (*N* = 5) and nonbreeding (*N* = 6; *p* < 0.001). Plasma levels of testosterone (**b**) were higher during breeding than during molt and nonbreeding (*p* = 0.01)

Testis volume was larger during breeding than during molt (*p* < 0.0001, Cohen’s d = 10.9) and nonbreeding (*p* < 0.0001, Cohen’s d = 11.1; overall F _(2, 15)_ = 323.03, *p* < 0.001 and *R*^2^ = 0.97; Fig. 3a).

Baseline plasma levels of testosterone were higher during breeding than during molt (*p* = 0.03, Cohen’s d = 1.6) and nonbreeding (*p* = 0.02, Cohen’s d = 1.5; overall F _(2, 15)_ = 6.41, *p* = 0.0097 and *R*^2^ = 0.4; Figs. 3b and 5). These baseline levels of testosterone did not differ from the second testosterone samples taken 30 min after capture, when the birds were decapitated (F _(1, 46)_ = 0.66; *p* = 0.42).

### Hormone receptor and aromatase mRNA expression

#### Hypothalamic and preoptic areas

The optical densities of aromatase (Pearson’s r = 0.87, *p* < 0.0001), androgen receptor (Pearson’s r =0.92, *p* < 0.0001), and estrogen receptor mRNA (Pearson’s r =0.64, *p* = 0.007) were correlated between the POA and the hypothalamus.

**Fig. 4:**
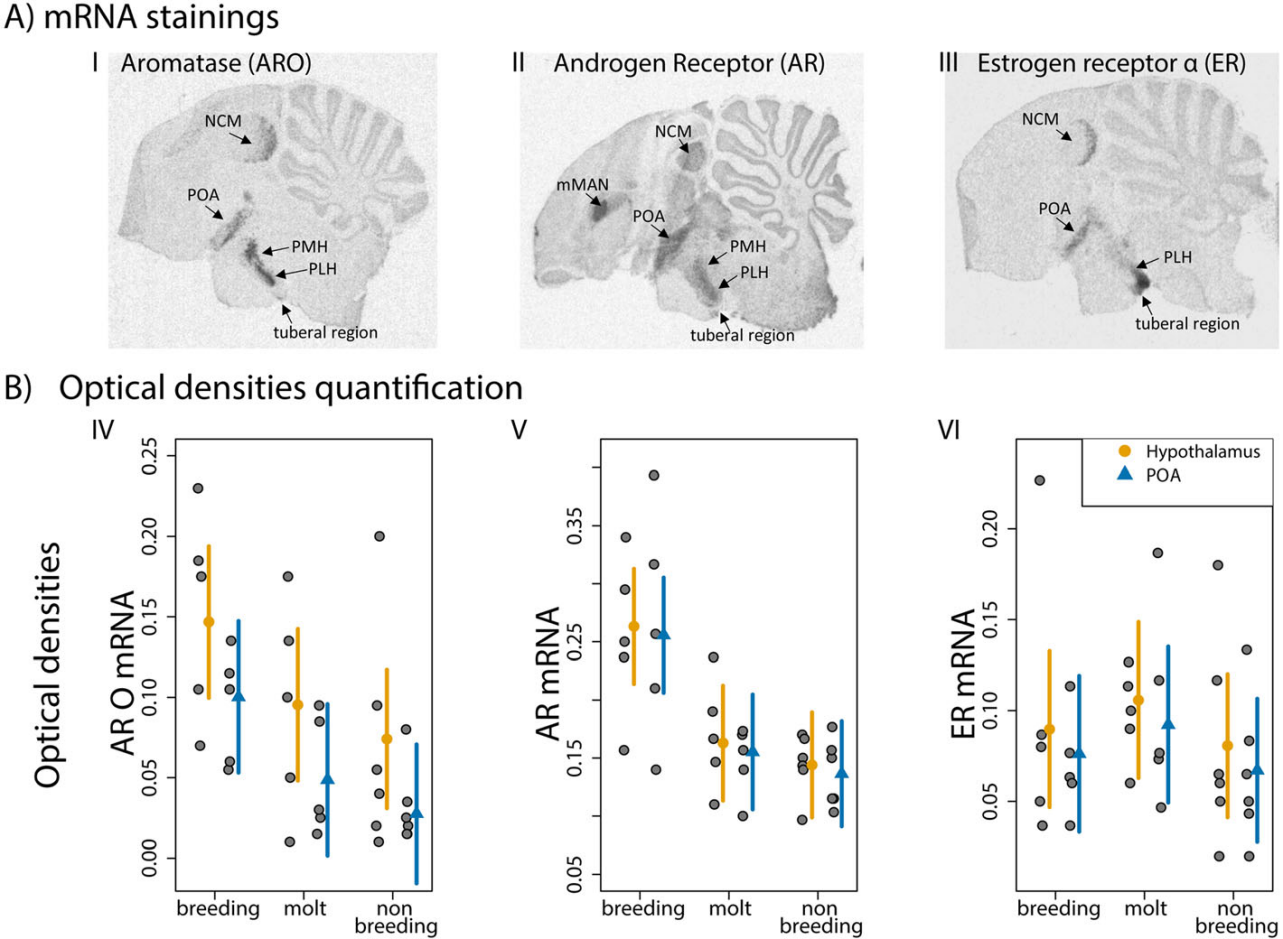
Seasonal analysis of hypothalamic brain areas. Example of slides (**a**) showing mRNA expression of aromatase (I), androgen receptor (II), and estrogen receptor α (III). NCM: caudo medial nidopallium, mMAN: medial nucleus magnocellularis of the anterior forebrain, POA: preoptic area, PMH: medial posterior nucleus of the hipothalamus, PLH: lateral posterior nucleus of the hypothalamus. Yellow and blue dots indicate mean estimates ± 95% CI, grey dots indicate individual data points. Aromatase (IV) and androgen receptor (V) mRNA expression were higher during breeding compared to the nonbreeding periods (*p* = 0.04, *p* = 0.01). Estrogen receptor (VI) mRNA expression in the preoptic area and the hypothalamus did not differ across seasons (breeding: *N* = 5, molt: *N* = 5, nonbreeding: *N* = 6)

**Fig. 5:**
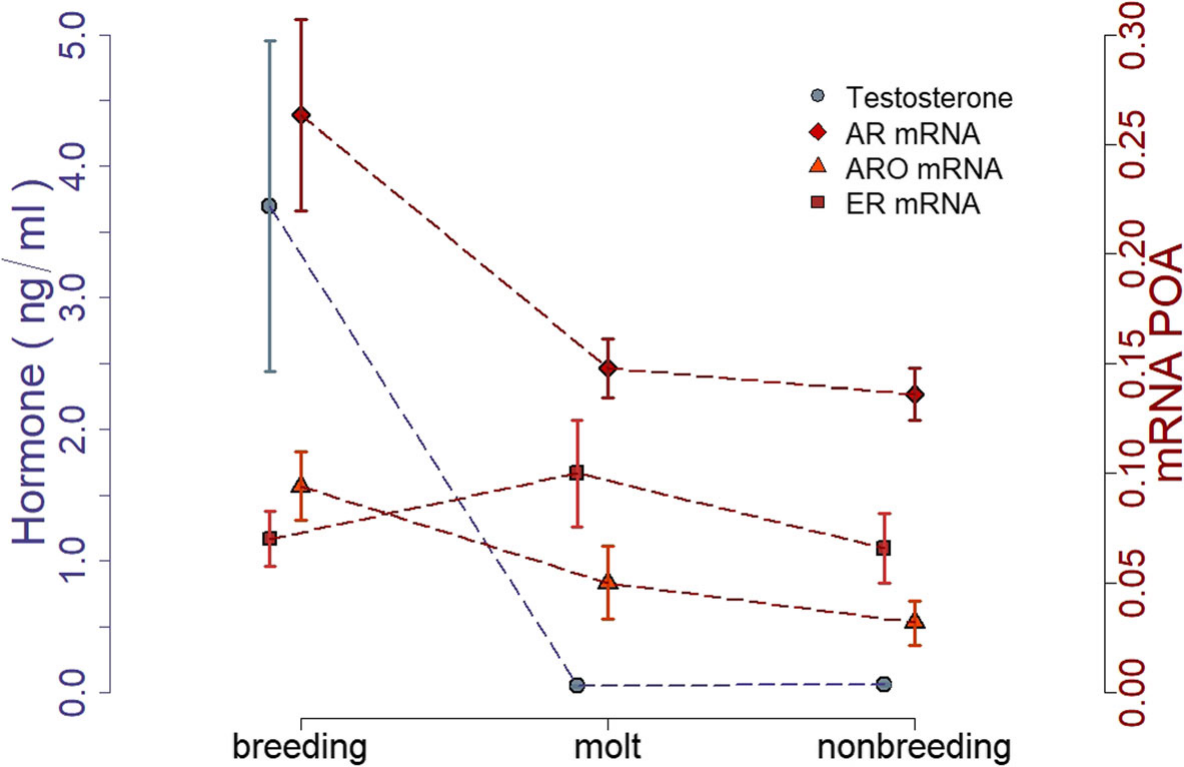
Summary of the seasonal hormone concentration and mRNA expression in the preoptic area. Points and bars indicate mean ± SE. Testosterone, androgen receptor and aromatase expression were higher during breeding compared to the nonbreeding periods. Estrogen receptor expression did not differ across seasons. Non-vocal territorial behavior was similar across all three stages

Aromatase mRNA expression differed between stages (χ^2^ =6.5, *p* = 0.04; Figs. 4IV and 5), it tended to be higher during breeding than during nonbreeding (*p* = 0.06, Cohen’s d: POA = 2.1, H = 1.2; Fig. 4IV) and was lower in the POA than the posterior hypothalamus (χ^2^ =13.4, *p* = 0.0002). Androgen receptor mRNA expression differed between stages (χ^2^ =13.3, *p* = 0.01), it was higher during breeding than during molt (*p* = 0.02, Cohen’s d: POA = 1.6, H = 1.5) and nonbreeding (*p* = 0.01, Cohen’s d: POA = 1.8, H = 2.1; Figs. 4V and 5). Within seasons, there was no difference between POA and hypothalamus (χ^2^ =0.6, *p* = 0.4). Estrogen receptor expression did not differ between stages (χ^2^ =1.3, *p* = 0.3; Figs. 4VI and 5), or between brain areas (χ^2^ =1.1, *p* = 0.6).

#### Song control system

**Fig. 6:**
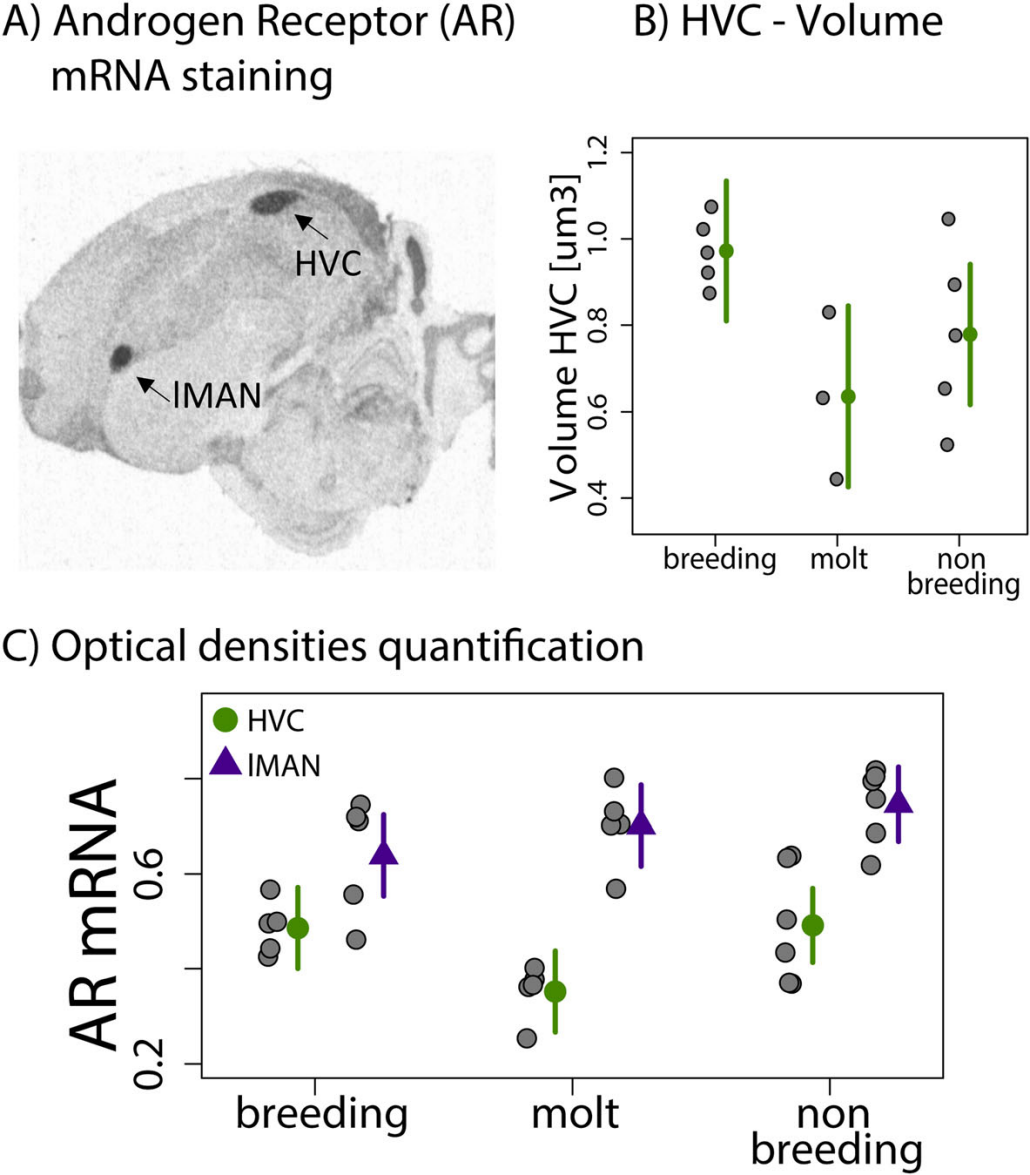
Seasonal analysis of song control areas. Androgen receptor mRNA expression (**a**). HVC: proper name, lMAN: lateral nucleus magnocellularis of the anterior forebrain. Green (HVC) and purple dots (lMAN) indicate mean estimates ± 95% CI, grey dots indicate individual data points. The volume (**b**) of the HVC was higher during breeding (*N* = 5) compared to the molt stage (*N* = 3, *p* = 0.04), but did not differ to nonbreeding (*N* = 6). Androgen receptor mRNA expression (**c**) in lMAN did not differ across seasons. There was a significant interaction effect between brain nuclei and seasons (*p* = 0.01), with androgen receptor expression in HVC being lower during molt compared to breeding and nonbreeding and to lMAN (breeding: *N* = 5, molt: *N* = 5, nonbreeding: *N* = 6). Males sung in response to the STI during breeding and nonbreeding, but not during molt

The volume of the HVC defined by androgen receptor mRNA (Fig. 6a) differed across seasons (F _(2, 10)_ = 4.28, *p* = 0.046, *R*^2^ = 0.35): it was larger during breeding than during molt (*p* = 0.05, Cohen’s d = 2.3), but did not significantly differ between breeding and nonbreeding (*p* = 0.27, Cohen’s d = 1.25) or between nonbreeding and molt (*p* = 0.7, Cohen’s d = 0.7, Fig. 6b). The volumes of the HVC measured by Nissl staining and defined by androgen receptor mRNA expression were correlated (F _(1, 11)_ = 354.2, *p* < 0.0001, *R*^2^ = 0.96).

The mRNA expression of androgen receptors of both nuclei in the song control system (Fig. 6a) did not differ between stages (χ^2^ =4.3, *p* = 0.1) and was lower in the HVC than in lMAN (χ^2^ =36.3, *p* < 0.001). There was a significant interaction between stage and brain area (χ^2^ =8.9, *p* = 0.01) resulting mainly from the molting stage (see Fig. 6c): while the mRNA expression of androgen receptors within lMAN did not differ across stages (breeding-molt: *p* = 0.9, Cohen’s d = 0.6; breeding-nonbreeding: *p* = 0.2, Cohen’s d = 1), the mRNA expression of androgen receptors in the HVC tended to be lower during molt than during breeding (*p* = 0.09, Cohen’s d = 2.4) and nonbreeding (*p* = 0.06, Cohen’s d = 1.4) and without difference between breeding and nonbreeding (*p* = 0.9, Cohen’s d = 0.05; Fig. 6c).

### Individual correlations

There was no correlation between circulating testosterone levels and expression of androgen and estrogen receptor mRNA or aromatase in the hypothalamic areas (all p’s > 0.1). Non-vocal territorial behaviors (latency to approach, time spent in 5-m radius, nodding frequency, and closest approach) did not correlate with circulating testosterone levels either (all p’s > 0.1; see supplementary Fig. 1 for details). Regarding the extent of non-vocal territorial behaviors (latency to approach, time spent in 5-m radius, nodding frequency, and closest approach) and androgen/estrogen receptor, or aromatase mRNA expression measured in the hypothalamic areas (POA and posterior hypothalamus) we found that the frequency of nodding behavior correlated positively with the expression of aromatase in the POA when corrected for stage (F_aro_=7.33, F_stage_ = 2.36, *p* = 0.02, *R*^2^ = 0.25). The time spent in a 5-m radius around the decoy was negatively correlated with the expression of estrogen receptors (F_er_=6.84, F_stage_ = 3.13, *p* = 0.02, *R*^2^ = 0.4) and the closest approach was positively related to the expression of estrogen receptors (F_er_=6.19, F_stage_ = 0.8, *p* = 0.03, *R*^2^ = 0.5) in the hypothalamus (see supplementary Fig. 3 for details). All other correlations between non-vocal territorial behaviors and androgen/estrogen receptors and aromatase mRNA expression were not significant (all p’s > 0.2). To account for autocorrelation, we did an effect size analysis, where no clear pattern was found in relation to either aromatase or estrogen receptor (see supplementary Fig. 2 for details). Song rate (number of songs sung 10 min after STI) was positively related to the optical density of androgen receptors in lMAN (deviance= 154.33 *p* < 0.001), negatively related to the aromatase expression in the POA (deviance= 166.40, *p* < 0.001) and positively related to the estrogen receptor expression in the POA (deviance=166.401, *p* < 0.001) and in the hypothalamus (deviance= 106.62, *p* < 0.001; see supplementary Fig. 4 for details). Finally, song type, song repertoire and syllable repertoire of part C were positively correlated to circulating testosterone levels (*p* = 0.02, *R*^2^ = 0.59, *p* = 0.02, *R*^2^ = 0.56; *p* = 0.03, *R*^2^ = 0.6).

## Discussion

Plasma testosterone concentrations and androgen receptor mRNA and aromatase mRNA expression changed seasonally in brain areas relevant for song (HVC and lMAN) and aggression (hypothalamic areas). In contrast, non-vocal territorial behaviors and estrogen receptor α expression in hypothalamic areas did not change across the three life-cycle stages; breeding, molt, and nonbreeding (autumn). Thus, our results suggest that non-vocal territorial behavior in black redstarts is decoupled from changes in circulating levels of testosterone and androgen receptor expression, confirming previous results from this species [8, 43, 44].

### Non-vocal territorial behavior

As expected, male black redstarts were territorial throughout all three life-cycle stages, even during molt when most bird species rarely show territorial behavior. Our results confirmed that seasonal changes in circulating levels of testosterone are not related to non-vocal territorial behavior (see also [8, 14, 16]). Yet, males only attacked the decoy during the breeding season and it is possible that males reacted more intensely to intrusions during breeding because they were guarding their mates. For male black redstarts in the studied population, mate-guarding may be essential to ensure reproductive success, given that a high percentage (33%) of nests contain extra-pair young [52]. Aromatase mRNA and androgen receptor mRNA showed a higher expression during the breeding season in the POA and the posterior hypothalamus (see Fig. 4). Both of these hypothalamic areas have been implicated in the control of sex-related and aggressive behaviors of birds [27–30, 53]. Previous studies have suggested that aromatase can regulate aggression via estrogen-dependent mechanisms outside the breeding season [20]. A further study proposed that dehydroepiandrosterone (DHEA) plays a role in the regulation of territorial behavior outside the breeding season, acting as a precursor for other active steroids such as estrogen or testosterone [54]. In black redstarts, however, the increased expression of aromatase mRNA in the POA during breeding did not reflect the rather constant territorial behavior seen in males across all life-cycle stages (Fig. 5). Indeed, previous work on black redstarts has already demonstrated that systemically applied aromatase inhibitors (letrozole) and androgen receptor blockers (flutamide) did not affect territorial behavior during and outside of the breeding season [43, 45]. Therefore, in black redstarts, it is unlikely that DHEA acts as a precursor of testosterone. The observed seasonal patterns of androgen receptor and aromatase expression in the hypothalamus are more likely to regulate mating behavior than aggressive behavior in males. On the other hand, estrogen receptor expression in both the POA and the posterior hypothalamus did not vary across seasons, but were negatively correlated with spatial territorial behaviors (supplementary Fig. 3). This would suggest a role of estrogen receptors in the regulation of territoriality and aggression. It has previously been shown that estradiol plays an important role in the behavior of male birds, including aggression and song [29, 47, 55, 56] even outside a breeding context [34, 57]. There may be several ways in which estrogens can affect territorial behavior: for example it may reduce or enhance auditory processing [58], it may be locally modulated in specific brain areas [55], or it may have rapid effects on aggression and on intracellular signaling [56]. In any case, the extent to which estrogen may affect territorial behavior in black redstarts needs further exploration, and would benefit from studies using larger sample sizes than ours. Future research might include experimental approaches with estrogen receptor blockers, or even consider other enzymes (5α and 5ß-reductase) that may regulate testosterone action [59]. Further, other brain areas should be involved in such analysis, since the neural circuits controlling territoriality and aggression of birds are not well known. Nevertheless, our results highlight the importance of exploring more deeply the relationship between non-vocal territorial behaviors and neuronal estrogen receptor expression in this species.

### Territorial song behavior

#### Song activity

Black redstarts showed marked seasonality in song: they sang in response to the STIs during the breeding and nonbreeding stages, but did not sing during molt (Fig. 2b). This seasonal pattern did not follow circulating levels of testosterone, which were high only during breeding (Fig. 3b), suggesting that the motivation and/or ability to sing must be independent of plasma testosterone. The seasonal pattern of song activity did, however, mirrored the changes in androgen receptor expression and the volume of the HVC, which were both increased during breeding and nonbreeding, when males actively sing, compared to the molt period when males did not sing (Fig. 6). The relationship between the seasonality of song and brain plasticity has been widely studied in captive birds [18, 23, 26, 60, 61]. For example, high levels of androgen receptor expression in HVC during breeding and nonbreeding, with low levels during molt, have also been observed in the domestic canary [23, 62], but similar patterns have been less frequently observed in free-living birds [7, 63–65]. HVC is indeed important in the control of the temporal organization of song [66] but might not be involved in singing activity beyond the observation that HVC needs a certain size or anatomical organization to enable birds to produce a song pattern [4, 62]. It remains to be seen whether the reduced androgen receptor sensitivity of HVC during molt might lead to a differentiated state of the song system that makes it impossible to organize a song pattern and thus precludes singing activity during this time. Previous studies indicated that the preoptic area is involved in the motivation of birds to sing, and thus likely influences seasonal song activity [30, 31, 67]. Yet, according to our results, seasonal patterns of androgen receptor mRNA and aromatase expression in the preoptic area did not correlate with changes in singing activity in black redstarts. Since changes in song rate were positively related to estrogen receptor expression in the POA and in the hypothalamus, and negatively associated to aromatase expression, it suggests that estrogens, but not testosterone, might modulate the motivation to sing [30, 40]. In addition, song rates correlated positively with androgen receptor expression in lMAN, a nucleus of the anterior forebrain pathway of the song control system that is involved in song plasticity in adult songbirds [68–70]. Androgen receptor expression in lMAN has been reported for several bird species [23], but the effect of androgen receptors on the role of lMAN in singing activity is unknown. As for HVC (see above), the androgen-dependent organization of local circuits might be necessary for lMAN to be functional. In the zebra finch, the firing mode of lMAN neurons depends on the social context and may contribute to adjusting the variability of the song pattern to the context [69]. However, we don’t know if song stereotypy of black redstarts changes between territorial and other contexts, e.g. during courtship. Future studies on black redstarts could further elucidate the mechanisms of singing activity in relation to motivational and physiological parameters in different life-cycle stages and in different competitive or breeding contexts.

#### Song structure

We found seasonal differences in the spectral features and in the song repertoire of the black redstart’s song. Concerning the spectral features, we found lower maximum frequencies of part A and B during breeding compared to nonbreeding (Fig. 2b and c). A previous study showed that male black redstarts treated with an androgen receptor blocker and an aromatase inhibitor changed the frequencies of part A and B of their song [43], which suggests an influence of androgens on those song features. In the current study it was only the maximum frequencies that differed between stages, suggesting a potential effect of circulating testosterone levels on this song parameter during breeding. Yet, we cannot rule out that the variations in maximum frequency are due to changes in sound amplitude, associated to the distance of the microphone and the direction in which the bird sings [71].

To assess the role of androgen receptor expression in the HVC on song structure, we performed a syllable repertoire analysis. In a previous study, we found more repetitive elements in part A and C of the black redstarts’ song during the breeding season [14]. Here we can add that the song syllable repertoire of part C also increases during the breeding season (Fig. 2g). Because the total number of syllables per song did not differ between breeding and nonbreeding (Fig. 2f), the augmentation of the song repertoire for part C was not due to a more complex song during breeding, but rather to a tendency of males to sing more song types (Fig. 2e). In other species, song features change between different life-cycle stages, for instance, the number and length of repetitive elements [64, 67, 72, 73] or the song repertoire size [74]. In black redstarts, although the reproductive context may be a major factor, the function of this increase in repertoire size of part C in response to the STI during spring needs further exploration. Unpublished data (C. Villavicencio) examining song maturation in black redstarts showed that first year males failed to match adults in the frequency bandwidth and maximum frequency of song part C, suggesting that this song part may indeed be most relevant in the breeding context. Changes in song structure can be seasonally mediated by circulating testosterone, and our results support this [4, 38, 75]. However, seasonal changes in androgen sensitivity of HVC are unlikely to be involved in repertoire changes, because androgen sensitivity did not differ between breeding and nonbreeding. Nevertheless, seasonally differential levels of testosterone would induce different HVC transcriptomes during breeding and nonbreeding [76, 77], which likely determine the functionality of HVC in the temporal control of song. In addition, the song structure might be influenced by the effect of high levels of testosterone on the syrinx muscles during breeding compared to nonbreeding [30], although such peripheral effects are unlikely to increase the number of syllable types in a subpart of the song as found in the black redstart.

## Conclusions

Our results suggest that high levels of circulating testosterone do not have seasonal activational effects on song or territorial behavior, because male black redstarts remain territorial for most of the year. They also sing during nonbreeding in autumn with basal levels of plasma testosterone. Circulating testosterone levels showed similar seasonal patterns as androgen receptor and aromatase expression in hypothalamic and preoptic areas, being higher during breeding and lower during molt and nonbreeding. These peaks in seasonal hormones and brain sensitivity were not associated with the expression of song and territorial behavior, suggesting that they may be exclusively associated with reproductive function and behavior. Yet, territorial and song behavior might be modulated to some extent by plasma testosterone, since higher circulating levels coincided with aggressive physical attacks and some qualitative changes in song features. On the other hand, the expression of estrogen receptors in the brain was related to territorial behavior, suggesting a possible modulatory effect of estradiol. Song activity vanished seasonally during the molt, in correlation with the reduced volume and androgen receptor mRNA expression of the HVC. This correlation suggests that androgen receptor expression in HVC may be related to seasonal singing activity, yet this do not explain differences in the song structure between seasons. Although song pattern changed slightly during the breeding season compared to the non-breeding season, this was not accompanied by changes in HVC volume and androgen receptor mRNA expression. In addition, considering that other authors propose that song motivation should be regulated by the preoptic area [30, 31], the relationship between seasonal singing activity and the seasonal expression of androgen receptor mRNA in these brain areas remains unclear in this species. Our studies of black redstarts have shown that there is no clear relationship between changes in neuronal expression of androgen receptors and aromatase with circulating testosterone levels and behavior. These findings might be related to the highly specific patterns of hormone regulation on behavior observed in songbirds, which include species-specific differences in genomic sensitivity to hormone receptors [38]. Our study suggests that the regulation of behavior and song across seasons and in different contexts might implicate multiple regulatory mechanisms, highlighting the importance of future work in assessing variation in behavior in relation to variation in brain sensitivity across seasons to better understand the complex comparative neuroendocrinology of free-living birds.

## Methods

### Experimental procedures

We compared the territorial and song behavior, plasma levels of testosterone, and brain sensitivity (expression of AR, ERα receptors and ARO) of adult male black redstarts during three different stages of the annual cycle: breeding (12th of April – 1st of May), molt (8th – 17th of August) and nonbreeding (2nd – 11th of October) in 2012. The study was carried out in Bavaria in the vicinity of the Max Planck Institute for Ornithology, Seewiesen.

We assessed the territorial behavior of males in response to simulated territorial intrusions (STIs) by placing a stuffed adult male decoy together with a playback of male songs into a male’s territory for 10 min. We continued to assess the song behavior of males for 10 min after the 10-min STI, because typically black redstarts only start singing once an STI is stopped. To avoid pseudoreplication, we used 3 different decoys and the songs of 20 different males unknown to the experimental subjects. We measured the latency time of males to respond to the STI, the time they spent in a 5-m radius around the decoy, and the frequency of head nodding; a sign of agitation in this species. Further, we quantified how closely they approached the decoy and recorded whether they physically attacked it. All the behaviors were quantified by the same person (CPV). We measured the territorial behavior of a total of 32 males: 9 during breeding, 10 during molt, and 14 during nonbreeding. During breeding and nonbreeding, we recorded the songs and quantified the song rate of 8 focal males per season both during the 10-min period of the STI and 10 min after the end of the STI. We continued to record the males until we had at least 20 full songs for each male (for some males a short playback was again used to stimulate the bird to sing). Recordings were made with a Sennheisser directional microphone (ME66/K6) connected to a Marantz solid state recorder PMD 660 (sampling frequency: 44.1 kHz; resolution: 16 bit).

After recording the aggressive and song behavior, we activated ground traps (~ 5) baited with mealworms to catch the focal male. Males were bled for testosterone immediately after capture (5.5±2.1 mean ± SD minutes); according to previous studies in this species, this time-lag did not affect baseline levels of testosterone [8, 16, 44]. Blood samples were stored on ice until returning from the field. After bleeding, each male was measured (body mass, lengths of the right tarsus, the wing and the tail, and width and height of the cloacal protuberance). During the molt period we scored the state of molt by assessing both wings and the feathers of 21 parts of the body. All birds were molting at least 4 feathers of each wing, and more than 62.5% of the body feathers. Approximately 30 min after the measurements were taken, males were killed by decapitation, the brain was rapidly extracted and immediately stored on dry ice in the field and in a − 80 °C freezer upon return to the laboratory. Directly after brain collection, both testes were extracted, measured and stored on dry ice together with the brain and the rest of the body. All samples were stored at − 80 °C. In total, we caught 18 of the 32 behaviorally tested males and obtained plasma, brain and testis samples from 7 breeding, 5 molting and 6 nonbreeding males. Due to technical problems during the brain sectioning, the brain slices of 2 breeding males were unavailable for in-situ hybridization, lowering the sample size to 5 brains for the breeding period. In addition, we did not collect the behavioral data for 1 of these 5 breeding males.

### Hormone analysis

Testosterone was analyzed by radioimmunoassay (RIA) following the protocol described by [78], and validated for this species [8, 14, 16, 44]. Hormone concentrations were calculated with Immunofit 3.0 (Beckmann Inc., Fullerton, CA, USA). Samples were measured in duplicate and distributed randomly between two assays. The extraction recovery was 84% ± 4.5% (mean ± sd). The lower detection limits of the testosterone assays were 3.7 pg/ml and all samples were above the detection limit. The intra-assay coefficient of variation of the testosterone standard was 4.1%; the intra-extraction coefficient of variation of a chicken plasma pool was 7.4%. The testosterone antibody cross-reacts with 5a-dihydrotestosterone; therefore, our measurements may include a minor fraction of this additional androgen.

### Song analysis

Song analyses were divided into spectral features and syllable repertoire. The former was performed in Avisoft-SAS Lab Pro software (version 4.25.196) and the latter in the custom written software in Delphi Pascal for Windows, Sound Explorer ([79], available at https://github.com/ornith). For the spectral features analysis spectrograms were used to visualize the recordings (sample rate 22,050 Hz, FFT =197,256 points, Hamming-window, overlap: 50%). A single strophe of a black redstart song constitutes three parts: A, B and C (see Fig. 2a, see also [45]). Parts A and C are each made up of a number of individual elements, whereas part B is classed as a single, atonal element. A pause of variable length separates parts A and B. The number of elements in parts A and C were determined using the automatic parameter measurements tool in Avisoft –SASLab (automatic single element separation; threshold: -20 dB, hold time: 5 ms). Some individuals sang a preceding string of short and repetitive trill-like elements at the beginning of Part A. In these individuals, these simple elements constitute a large part of the overall number of elements in part A.

The 20 best quality strophes (low background noise) from each individual were selected to analyze several song parameters. The maximum and minimum frequencies, total frequency bandwidth and the duration was measured for each part (A, B and C) of each individual strophe, as well as the duration of the pause between parts A and B and the total duration and overall frequency bandwidth of each entire strophe.

The same best quality songs were used for the syllable repertoire analysis. First, the songs were merged in one file for each individual and sounds were extracted using an amplitude trigger level set by the user. Songs were manually cleaned (denoised, high/low frequency pass filtered) if other sounds (e.g., calls from other birds, wind) prevented the correct syllables’ separation, set at 10 ms. The sounds were converted into sonograms assembled from 256 points fast Fourier transforms (Intel libraries). From the sonograms the average frequency, modal frequency, fundamental frequency (first peak), Wiener entropy, duration, and their standard deviations were calculated and the sonograms were subsequently clustered. Sorting was done using a k-means clustering algorithm, starting with two clusters and splitting new clusters off one at a time. After clustering, every cluster was viewed and mistakes were corrected based on visual inspection. From this analysis, we measured for each individual the number of song types (when singing, black redstarts alternate between different song types: each type has a stereotyped sequence of elements and the whole song type can be repeated several times), the total number of syllable types (hereafter repertoire size), the repertoire size for each part (part A and part C; part B was excluded because it is always constituted of a single atonal element), and the total number of syllables per song for each bird, during spring (*N* = 8) and nonbreeding (*N* = 7; total songs=320).

### Brain analysis

Frozen brains were cut into 20 μm sagittal sections on a cryostat microtome (Leica Microsystems GmbH, Wetzlar, Germany) and collected on superfrost object slides (Menzel GmbH, Braunschweig, Germany) in 10 parallel series [7, 65]. From each of the parallel series, the first brain section was selected for in situ hybridization (see below for details) of androgen receptor (AR) mRNA, the second adjacent series was selected for estrogen receptor α (ER α) mRNA, the third adjacent series for aromatase (ARO) mRNA expression, and the fourth adjacent series for Nissl staining. Thus, in each staining procedure the interval between sections was 200 μm. Autoradiograms were scanned with an Epson scanner using SilverFast Ai software as 16-bit grey values and with a resolution of 2400 dpi for later analysis in ImageJ. Optical densities of androgen receptor mRNA expression levels were individually measured in the HVC and lMAN. For both, we used a square with fixed dimensions (0.2 × 0.2 mm) that was positioned in the middle of the HVC and lMAN area of every second section that included those nuclei (~ 10 sections for HVC and ~ 6 sections for lMAN; see [7]). The volume of HVC was estimated based on the androgen receptor expression and on the Nissl stained samples [27]. For each androgen receptor-labeled and Nissl stained brain section we delineated HVC, summed the area measured, and multiplied them with 200 μm (i.e. the distance between subsequent sections analyzed for the same receptor/enzyme; [7]; [65]). To estimate volumes in each individual, the HVC was delineated and calculated three times, then the measurements were averaged to obtain a final individual HVC volume. All measurements were carried out by one person (RQ), blind to the identity of the sections. The volume of RA and of lMAN were not measured. In case of RA, the androgen receptor expression covered the whole archopallium so that delineation of RA was uncertain. Given that lMAN is not part of the motor pathway of the song control system, we decided not to include measurements of volumes associated with seasonal song production. Optical densities of mRNA expression of aromatase, androgen receptor and estrogen receptor α were individually measured in the preoptic area and in the posterior hypothalamus (see Fig. 4) in the following way: the measurements were taken within the whole delineated areas of the preoptic area and of posterior hypothalamus. The whole posterior hypothalamus measured included the nucleus posteriori hipothalamic medialis (PMH), nucleus posteriori hipothalamic lateralis (PLH) and the tuberal region. We delineated the borders based on the mRNA receptor or enzyme expression in all sections that included the areas (2–3 sections), with sizes differing between individuals (Fig. 4, see also [14]). To control for background staining, the optical density of a control area just adjacent to the area measured was subtracted from the value for receptor expression [14]. Optical density measurements were averaged across all sections.

#### In-situ hybridization

Riboprobes were synthesized from cDNA previously cloned from zebra finch androgen mRNA [23]. Antisense and sense 35S-CTP-labeled probes were transcribed from the T7 and SP6 promoter region of a pGEM7Zf + vector using the Riboprobe System (Promega, Madison, WI). Brain sections were fixed in 4% formaldehyde in phosphate-buffered saline (PBS; 0.01 M; pH 7.4) for 5 min, washed in DEPC-treated PBS, and incubated in 0.25% acetic anhydride in ethanolamine (TEA; 0.1 M; pH 8.0) for 10 min to reduce nonspecific binding. After a washing step in 2x standard saline citrate (SSC), sections were dehydrated in serially increasing percentages of ethanol, and left to dry at room temperature. Sections were hybridized under a cover slide with 35S-CTP-labeled sense or antisense riboprobes (0.4 × 106 cpm/slide) in hybridization buffer with 50% formamide and 10% dextran sulfate overnight at 55 °C. After hybridization, slides were immersed in 2x SSC at room temperature to remove the cover slides and incubated in RNase A (20 μg/ml) for 30 min at room temperature. Sections were then consecutively washed for 30 min in 2x SSC at 50 °C, 0.2x SSC at 55 °C, and 0.2x SSC at 60 °C, dehydrated in ethanol containing 0.3 M ammonium acetate, and dried for 1 h at room temperature. All the slides used with brain sections were processed in two separate groups. Finally, slides were exposed to Kodak BioMax MR film (Sigma–Aldrich Co., St. Louis, MO) in lightproof boxes for 3 weeks at room temperature, developed in Kodak D-19 developer, washed in tap water, and fixed with Kodak fixer.

### Data analysis

All data were analyzed using R 3.5.0 (R Development Core Team), the package “arm” [80] and the package “emmeans” [81]. Two kinds of analyses were done. First, to assess differences across seasons we used general linear models with vocal and non-vocal territorial behaviors and testosterone as dependent factor and stage as independent factor. To assess seasonal differences of androgen receptor, estrogen receptor and aromatase mRNA expression we used linear mixed models with mRNA expression as the response variable, brain area (POA and H or HVC and lMAN) and season as independent factors, and male ID as random effect to account for the repeated measures, since we measured the different brain areas of each male. In addition, the correlation of hormone receptor expression between brain areas was done using Pearson’s correlation. For the song analysis, we analyzed the frequency of the total songs including complete and incomplete songs (incomplete songs mainly consisted of just part A, or parts B and C sung together) sang 10 min after STI with stage as independent factor. Then, all the song parameters were analyzed using linear mixed models with stage as a factor and the male ID as random effect to account for repeated measures (as we had 20 songs per male). For the syllable repertoire analyses we used general linear models for the song type, the song repertoire, and the number of syllables per song, with stage as factor. To analyze the number of syllables per part per bird we used a linear mixed model with stage and part as factor and the male ID as random effect to account for repeated measures. We used post-hoc analysis with Bonferroni correction and false discovery rate (FDR) analysis when necessary. Second, to assess the individual correlations of behavior, hormones and brain mRNA receptors and aromatase mRNA expression we used linear models. We used behavior as response variable and hormone levels or receptor and aromatase mRNA expression as covariates and stage as independent factor. The behavioral variables were not highly correlated, and the sample size was low for performing a principal component analysis. Therefore, to account for autocorrelation, we did an effect size analysis. In addition, we used brain receptor and aromatase mRNA expression as response variable, hormone levels as covariate and stage as independent factor. To relate the song frequency with receptor and aromatase mRNA expression or hormone concentrations we used generalized linear models with “Poisson” distribution and stage as independent factor. For all models with two factors, we assessed the interaction between season and the correspondent factor (e.g brain area or testosterone) in all initial models, however, if the interaction was not significant, we removed it from the model. Residuals were checked to see if model assumptions were met. Variables were square-root or log transformed if needed to fulfill model assumptions (time spent in a five-meter radius around the decoy and closest approach).

## Supplementary Information

**Additional file 1: Supplementary Figure 1***:* Pearson correlation coefficients and their 95% confidence intervals as a measure of standardized effect sizes of all aggressive traits measured in relation to testosterone concentrations. Positive coefficients indicate positive association of the respective behaviour with testosterone. All effects were slightly positive, but the 95% confidence intervals included zero, suggesting that the degree of aggressiveness was not strongly associated testosterone (t5m: time in 5-m radius around the decoy, lat: latency to approach the decoy, nod: frequency of head nodding, ca: closest approach to decoy, song: song rate). To be consistent with the other behaviors, the latency to approach and the closest approach are represented with a negative sign, because smaller latencies and closer approaches indicate higher aggression, whereas for all other behaviors larger values indicate higher aggression. **Supplementary Figure 2***:* Pearson correlation coefficients (± 95% confidence intervals) of all aggressive behavioral traits measured in relation to aromatase, androgen receptor and estrogen receptor expression in the preoptic area (POA, top row panels) and hypothalamic areas (H, lower row panels). Correlation coefficients were consistently positive for aggressive behaviours in relation to aromatase expression in the hypothalamus, but the 95% confidence intervals included zero in all of the measures, suggesting that there was no strong relationship between aggression and aromatase expression in the hypothalamus. The correlation coefficients of all other measures in both brain areas included negative and positive values suggesting no consistent association with aggressive behavior (see Supplementary Fig. 1 for further information). **Supplementary Figure 3***.* Correlation between territorial behaviors in response to an STI and brain aromatase expression in the preoptic area and estrogen receptor expression in the hypothalamus. The nodding frequency correlated positively with the expression of aromatase in the preoptic area when corrected for stage (F_aro_=7.33, F_stage_ = 2.36, *p* = 0.02, *R*^2^ = 0.25; Suppl. Fig. 3A). The time spent in a 5-m radius around the decoy was negatively correlated with the expression of estrogen receptors (F_er_=6.84, F_stage_ = 3.13, *p* = 0.02, *R*^2^ = 0.4) and the closest approach was positively related to the expression of estrogen receptors (F_er_=6.19, F_stage_ = 0.8, *p* = 0.03, *R*^2^ = 0.5) in the hypothalamus (Suppl. Fig. 3B). **Supplementary Figure 4***:* Song rate in relation to brain sensitivity. The song frequency was positively related to the optical density of AR in lMan (deviance= 154.33 *p* < 0.001), song frequency was negatively related to the aromatase expression in the POA (deviance= 166.40, *p* < 0.001) and positively related to estrogen receptor expression in the POA (deviance=166.401, *p* < 0.001) and in the H (deviance= 106.62, *p* < 0.001).

## Data Availability

The datasets generated and/or analyzed during the current study are available in the Edmond repository (Open Research Data Repository of the Max Planck Society), [ https://dx.doi.org/10.17617/3.5e].
