# Supplementary material for: Neuroendocrine patterns underlying seasonal song and year-round territoriality in male black redstarts
Source: Front Zool. 2021 Feb 24;18:8. doi: 10.1186/s12983-021-00389-x (PMC7905601; doi:10.1186/s12983-021-00389-x)
Supplement: Supplementary file 1 — Additional file 1: Supplementary Figure 1: Pearson correlation coefficients and their 95% confidence intervals as a measure of standardized effect sizes of all aggressive traits measured in relation to testosterone concentrations. Positive coefficients indicate positive association of the respective behaviour with testosterone. All effects were slightly positive, but the 95% confidence intervals included zero, suggesting that the degree of aggressiveness was not strongly associated testosterone (t5m: time in 5-m radius around the decoy, lat: latency to approach the decoy, nod: frequency of head nodding, ca: closest approach to decoy, song: song rate). To be consistent with the other behaviors, the latency to approach and the closest approach are represented with a negative sign, because smaller latencies and closer approaches indicate higher aggression, whereas for all other behaviors larger values indicate higher aggression. Supplementary Figure 2: Pearson correlation coefficients (± 95% confidence intervals) of all aggressive behavioral traits measured in relation to aromatase, androgen receptor and estrogen receptor expression in the preoptic area (POA, top row panels) and hypothalamic areas (H, lower row panels). Correlation coefficients were consistently positive for aggressive behaviours in relation to aromatase expression in the hypothalamus, but the 95% confidence intervals included zero in all of the measures, suggesting that there was no strong relationship between aggression and aromatase expression in the hypothalamus. The correlation coefficients of all other measures in both brain areas included negative and positive values suggesting no consistent association with aggressive behavior (see Supplementary Fig. 1 for further information). Supplementary Figure 3. Correlation between territorial behaviors in response to an STI and brain aromatase expression in the preoptic area and estrogen receptor expression in the hypothalamus. The nodding frequency corr [file 12983_2021_389_MOESM1_ESM.docx]

SUPPLEMENTARY MATERIAL


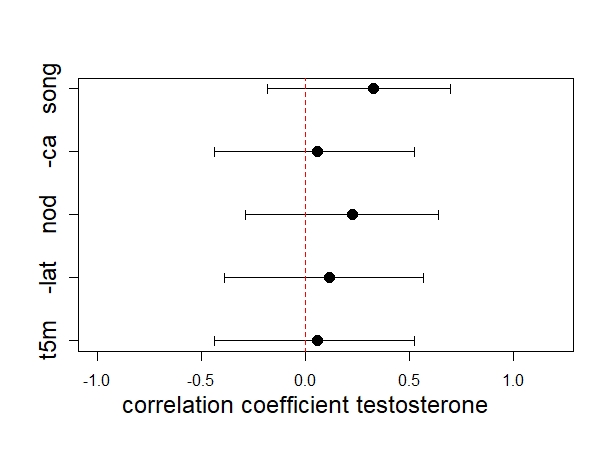


*Supplementary Fig. 1: Pearson correlation coefficients and their 95% confidence intervals as a measure of standardized effect sizes of all aggressive traits measured in relation to testosterone concentrations. Positive coefficients indicate positive association of the respective behaviour with testosterone. All effects were slightly positive, but the 95% confidence intervals included zero, suggesting that the degree of aggressiveness was not strongly associated testosterone (t5m: time in 5-meter radius around the decoy, lat: latency to approach the decoy, nod: frequency of head nodding, ca: closest approach to decoy, song: song rate). To be consistent with the other behaviors, the latency to approach and the closest approach are represented with a negative sign, because smaller latencies and closer approaches indicate higher aggression, whereas for all other behaviors larger values indicate higher aggression.*
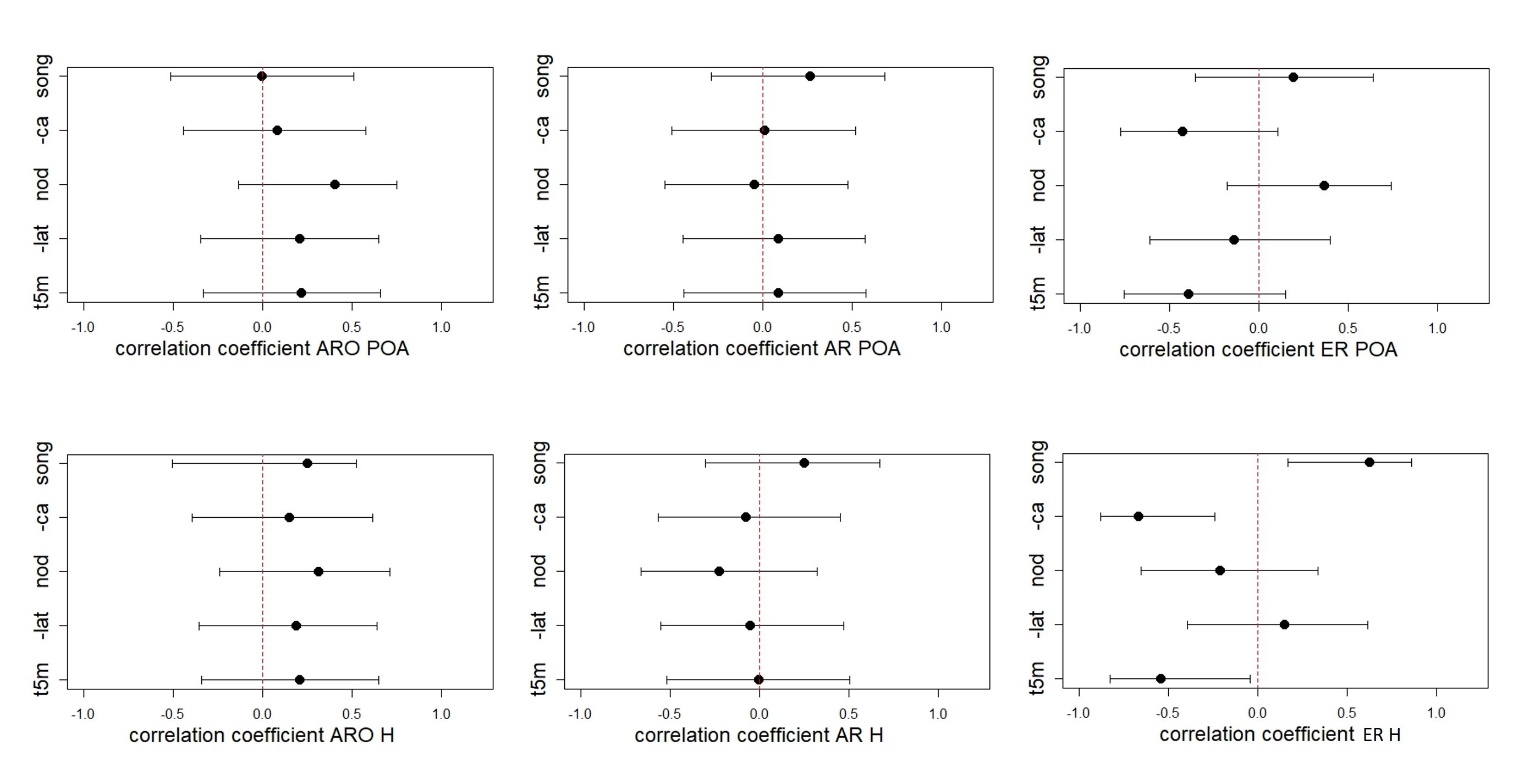


*Supplementary Fig. 2: Pearson correlation coefficients (± 95% confidence intervals) of all aggressive behavioral traits measured in relation to aromatase., androgen receptor and estrogen receptor expression in the preoptic area (POA, top row panels) and hypothalamic areas (H, lower row panels). Correlation coefficients were consistently positive for aggressive behaviours in relation to aromatase expression in the hypothalamus, but the 95% confidence intervals included zero in all of the measures, suggesting that there was no strong relationship between aggression and aromatase expression in the hypothalamus. The correlation coefficients of all other measures in both brain areas included negative and positive values suggesting no consistent association with aggressive behavior (see Supplementary Fig. 1 for further information)*


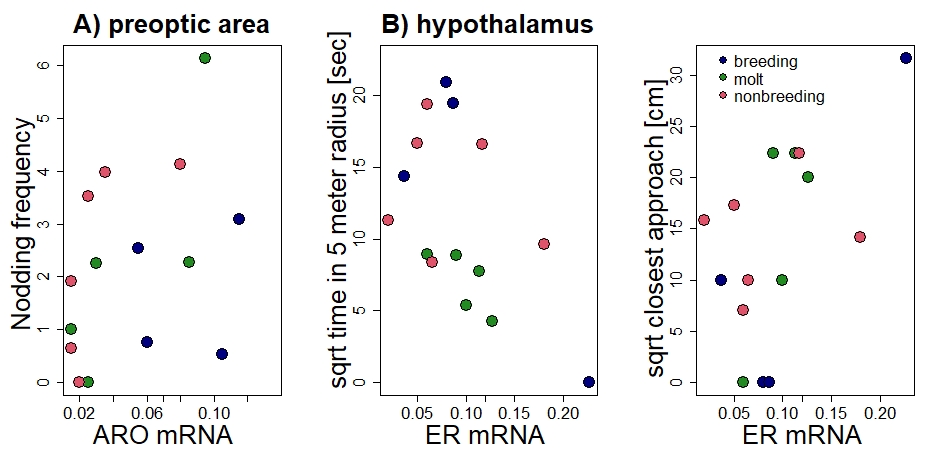


*Supplementary Fig. 3. Correlation between territorial behaviors in response to an STI and brain aromatase expression in the preoptic area and estrogen receptor expression in the hypothalamus. The nodding frequency correlated positively with the expression of aromatase in the preoptic area when corrected for stage (F_aro_=7.33, F_stage_ = 2.36, p=0.02, R^2^ = 0.25; Suppl. Fig. 3A). The time spent in a 5-meter radius around the decoy was negatively correlated with the expression of estrogen receptors (F_er_=6.84, F_stage_ = 3.13, p=0.02, R^2^ = 0.4) and the closest approach was positively related to the expression of estrogen receptors (F_er_=6.19, F_stage_ = 0.8, p=0.03, R^2^ = 0.5) in the hypothalamus (Suppl. Fig. 3B).*

**
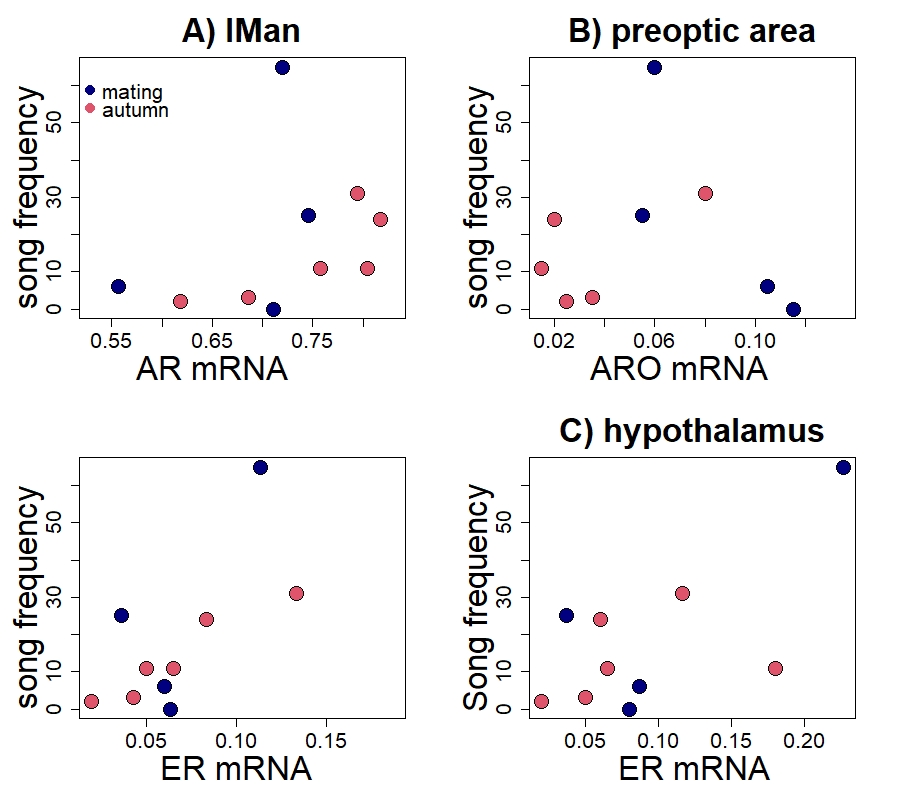
**

*Supplementary Fig. 4: Song rate in relation to brain sensitivity. The song frequency was positively related to the optical density of AR in lMan (deviance= 154.33 p < 0.001), song frequency was negatively related to the aromatase expression in the POA (deviance= 166.40, p < 0.001) and positively related to estrogen receptor expression in the POA (deviance=166.401, p < 0.001) and in the H (deviance= 106.62, p < 0.001).*
